# Characterization and comparative analysis of the complete mitochondrial genome of *Azygia hwangtsiyui* Tsin, 1933 (Digenea), the first for a member of the family Azygiidae

**DOI:** 10.3897/zookeys.945.49681

**Published:** 2020-07-03

**Authors:** Yuan-An Wu, Jin-Wei Gao, Xiao-Fei Cheng, Min Xie, Xi-Ping Yuan, Dong Liu, Rui Song

**Affiliations:** 1 Hunan Fisheries Science Institute, Changsha 410153, China Hunan Fisheries Science Institute Changsha China

**Keywords:** gene arrangement, phylogenetic analysis, Trematoda

## Abstract

*Azygia hwangtsiyui* (Trematoda, Azygiidae), a neglected parasite of predatory fishes, is little-known in terms of its molecular epidemiology, population ecology and phylogenetic study. In the present study, the complete mitochondrial genome of *A. hwangtsiyui* was sequenced and characterized: it is a 13,973 bp circular DNA molecule and encodes 36 genes (12 protein-coding genes, 22 transfer RNA genes, two ribosomal RNA genes) as well as two non-coding regions. The A+T content of the *A. hwangtsiyui* mitogenome is 59.6% and displays a remarkable bias in nucleotide composition with a negative AT skew (–0.437) and a positive GC skew (0.408). Phylogenetic analysis based on concatenated amino acid sequences of twelve protein-coding genes reveals that *A. hwangtsiyui* is placed in a separate clade, suggesting that it has no close relationship with any other trematode family. This is the first characterization of the *A. hwangtsiyui* mitogenome, and the first reported mitogenome of the family Azygiidae. These novel datasets of the *A. hwangtsiyui* mt genome represent a meaningful resource for the development of mitochondrial markers for the identification, diagnostics, taxonomy, homology and phylogenetic relationships of trematodes.

## Introduction

The genus *Azygia* Looss, 1899 is an endoparasitic helminth found in the stomach and intestine of freshwater feral carnivorous fish (Frolova and Shcherbina 1975). This genus includes several species complexes and its type species is *Azygia lucii* (Müller, 1776), which is a parasite of numerous, but especially esocid and percid, fishes in Europe. Many researchers have added to our knowledge of this cosmopolitan species. To date, species of *Azygia* are frequently reported from the esophagus, stomach, and intestine of a wide range of predatory fishes from Asia, Europe and North America, including China, Japan, India, Russia, Germany, and North America (Tubangui 1928; Tsin 1933; Moravec and Sey 1989; Marcogliese and Cone 1996; Besprozvannykh 2005; Jadhav et al. 2011; Pallewad et al. 2015; Womble et al. 2016; Nagasawa and Katahira 2017).

*Azygia hwangtsiyui* Tsin, 1933 is a member of the family Azygiidae Odhner, 1911 and is often overlooked; it is parasitic in the gastrointestinal tract of species of the family Channidae Fowler, 1934 but caused only slight clinical signs, including malnutrition and weight loss. In China, *Azygia hwangtsiyui*-infected freshwater predatory fishes have been described from Shandong, Heilongjiang, Jiangsu, Fujian, Sichuan and Hunan Provinces (Tsin 1933; Zmejev 1936; Ma 1958; Tang and Tang 1964; Kiang 1965; Chen 1973; Wang 1985; Cheng 2011). It has a mainly inland distribution and utilizes freshwater snail species (e.g. *Vivipara quadrata* (Benson, 1842)) as intermediate hosts (Tang and Tang 1964) and develops into adults in the gastrointestinal tract of predatory fish species such as *Ophiocephalus argus* Cantor, 1842 and *Channa asiatica* (Linnaeus, 1758) (Tsin 1933; Besprozvannykh 2005).

Morphology is the most commonly used method for species identification and differentiation of metazoans and is widely adopted globally by parasitologists and taxonomists. A huge disadvantage of using morphological criteria, however, is that it is difficult to identify and distinguish closely related and cryptic species. Although the family Azygiidae was erected more than a century ago, its situation, and that of several species of *Azygia*, is still controversial and uncertain. Manter (1926) pointed out that *Azygia* is the only genus in the family then presenting systematic confusion: *Azygia longa* (Leidy, 1851) in North America may be a synonym of *A. lucii* in Europe (Manter 1926), and Van Cleave and Mueller (1934) reported that *Azygia acuminata* Goldberger, 1911 and *A. longa* should be considered conspecific. Nevertheless, due to the discovery of some life histories of members of *Azygia*, *A. lucii* and *A. longa* have been recognized as two distinct species (Szidat 1932; Sillman 1953; Sillman 1962).

Mitochondrial (mt) genome and nuclear ribosomal DNA sequences are effective molecular tools for taxonomic identification, phylogeny and biogeographical research (Bernt et al. 2013; Le et al. 2019). However, only a partial cytochrome oxidase subunit 1 protein sequence (AIY67834) of *Proterometra macrostoma* Horsfall, 1933 (Azygiidae) is currently available in GenBank. None of the mitochondrial genome data have been sequenced for a member of the family Azygiidae. Therefore, we determined the complete mitochondrial genome sequence of *A. hwangtsiyui* as a basis for the future definition of strain- and species-specific markers, and for assessing mitogenomics in resolving the interrelationships of trematodes.

## Materials and methods

### Sampling and DNA extraction

The specimens of flatworms were isolated from the stomach of their definitive host, in this case snakehead fish (*Ophiocephalus argus* (Cantor, 1842)) obtained from east Dongting Lake in Yueyang, Hunan province, China (29°22'N, 113°06'E). *Azygia hwangtsiyui* was morphologically identified according to the original and other descriptions (Tsin 1933; Tang and Tang 1964; Zhang et al. 1999; Besprozvannykh 2005), using a stereomicroscope and a light microscope. Furthermore, single samples were confirmed molecularly as *A. hwangtsiyui* based on sequencing of 1370 bp 28S rDNA sequence. The parasites were completely washed in water, preserved in 99% ethanol, and stored at 4 °C until genomic DNA extraction. Total genomic DNA extraction was performed from an intact specimen with the TIANamp Micro DNA Kit (Tiangen Biotech, Beijing, China), according to the manufacturer’s instructions.

### DNA amplification and sequencing

According to conserved regions of mitochondrial genes in other available digenea mitogenomes, six partial gene fragments for cytb, nad4, nad1, 16S, 12S and cox2 were amplified using six generic primers sets HWF1/HWR1 (for cytb), HWF3/HWR3 (for nad4), HWF5/HWR5 (for nad1), HWF7/HWR7 (for 16S), HWF9/HWR9 (for 12S), and HWF11/HWR11 (for cox2), respectively. On the basis of these obtained nucleotide sequences, *A. hwangtsiyui*-specific primers were designed for amplification and sequencing of the remaining mitogenome (Suppl. material 1: Table S1). All primers were designed to produce amplicons with overlaps of approximately 100 bp. PCR reactions were performed in a 50 µl reaction solution with the ingredient of 18.5 µl ddH_2_O, 25 µl 2×PCR buffer (Mg^2+^, dNTP plus, Takara, Dalian, China), 1.5 µl of each primer (0.2–1.0 μM), 1 µl EX Taq polymerase (250U, Takara), and 2.5 µl DNA template. PCR amplification was compliant to the following amplification protocol: initial denaturation at 98 °C for 2 min, followed by 40 cycles 10 s at 98 °C, 15 s at 50 °C, 68 °C for 1 min/kb, and 10 min at 68 °C for a final extension. The amplified PCR products were purified with TIANgel Purification Kit (Tiangen Biotech, Beijing, China), and sequenced bidirectionally at Sangon Biotech (Shanghai) Co., Ltd. (Shanghai, China) based on the primer walking method using several specific primers (Suppl. material 1: Table S1).

### Mitogenome annotation and analysis

According to sequence chromatograms, all raw fragments were quality-proofed using CHROMAS (https://www.technelysium.com.au) to remove ambiguity codes and low-quality bases. Whenever the quality was sub-optimal, sequencing was repeated until the amplicon is the consensus sequence. Before manual assembly of the entire mitochondrial genomic sequence, identification of all amplicons was performed by BLASTN check (Altschul et al. 1990). The mt genome of *A. hwangtsiyui* was aligned against the mt genome sequences of other promulgated digenean mitogenomes utilizing multiple sequence alignment software MAFFT version 7.149 (Katoh and Standley 2013) to identify genetic boundary. Protein-coding genes (PCGs) were predicted with Open Reading Frame Finder (https://www.ncbi.nlm.nih.gov/orffinder/) adopting echinoderm and flatworm mitochondrial codes, and examining the nucleotide alignment against the reference mtDNA in trematode *Dicrocoelium chinense* Tang et Tang, 1978 (NC_025279.1). Whole tRNAs were inferred with the detection results of ARWEN (Laslett and Canback 2008) and MITOS web server (Bernt et al. 2013). Two rRNA (rrnL and rrnS) were founding by comparison with those of published fluke mitogenomes. Codon usage and relative synonymous codon usage (RSCU) for 12 PCGs of *A. hwangtsiyui* were computed by PHYLOSUITE (Zhang et al. 2020), and its operation results were imported into GGPLOT2 program (Wickham 2016) to make figures of the RSCU. Tandem repeats in the non-coding regions were determined with Tandem Repeats Finder software version 4.09 (Benson 1999), and the prediction of their secondary structures were performed by the MFOLD web server (Zuker 2003). The annular diagram of *A. hwangtsiyui* mitogenome was plotted with mitochondrial genome data visualization tool MTVIZ (http://pacosy.informatik.uni-leipzig.de/mtviz/mtviz).

### Phylogenetic analysis

For phylogenetic analyses, we utilized translated and concatenated amino acid sequences of twelve protein-coding genes for 49 Platyhelminthes including *A. hwangtsiyui* mitogenome determined in this study. Two tapeworm species, *Cloacotaenia megalops* (Nitzsch in Creplin, 1829) (NC_032295.1) and *Dibothriocephalus latus* (Linnaeus, 1758) (NC_008945.1) were included as outgroup taxa representing two different families. Species information including systematic positions and GenBank accession numbers is provided in Suppl. material 2: Table S2. The PHYLOSUITE program was used to extract twelve PCGs from the GenBank files, export fasta files with translated amino acid datasets, and align datasets in bulk using integrated applet MAFFT with normal-alignment mode. Phylogenetic analyses were performed using Bayesian Inference (BI) and Maximum Likelihood (ML) methods. Assessment of the best-fit evolutionary model for dataset was conducted via ModelGenerator v0.8527 (Keane et al. 2006). BI in MrBayes version 3.2.6 (Ronquist et al. 2012) was carried out under the MtRev matrix of amino acid substitution, and was analyzed with 1 × 10^7^ metropolis-coupled Monte Carlo Markov Chain (MCMC) generations. Two independent runs with four simultaneous MCMC chains (one cold and three heated chains) were conducted for 1 × 10^7^ million generations, sampling every 10,000 generations and discarding the initial 25% generations as burn-in. ML analysis in PHYLOSUITE was performed using MtART+I+G matrix with 1000 bootstrap replicates.

## Results and discussion

### General traits of the *Azygia hwangtsiyui* mitogenome

**Figure 1.**
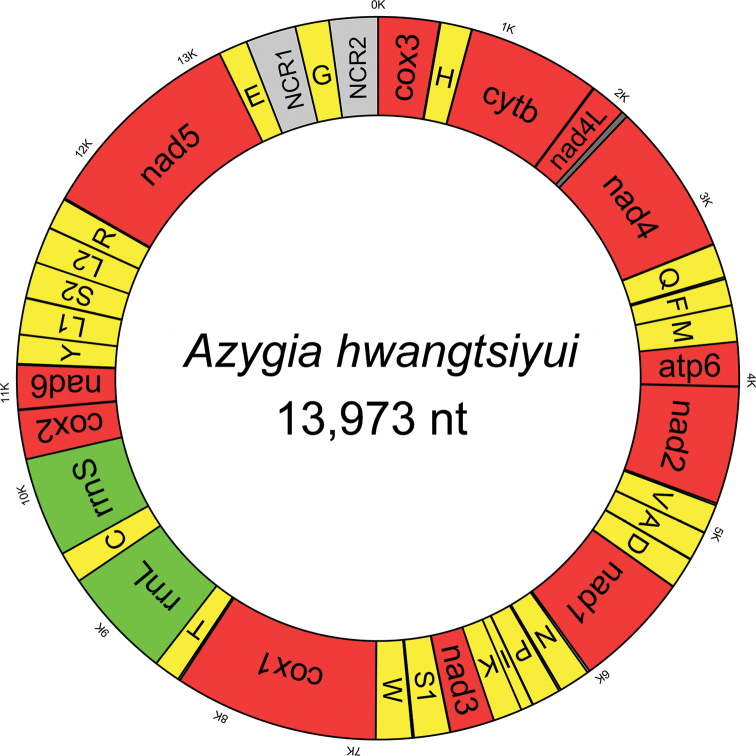
An annular diagram of the *Azygia hwangtsiyui* mitochondrial genome.

The entire *A. hwangtsiyui* mtDNA is 13,973 bp in length (GenBank accession number: MN844889) and comprised of 12 protein-coding genes (cox1-3, nad1-6, nad4L, cytb, and atp6), 22 tRNA genes, two rRNA genes (rrnL and rrnS), and two non-coding regions. The 12 protein-coding gene order arrangement is cox3-cytb-nad4L-nad4-atp6-nad2-nad1-nad3-cox1-cox2-nad6-nad5 (Fig. 1), which is identical to those of *Clinostomum complanatum* (Rudolphi, 1814), *Echinostoma hortense* Asada, 1926, and some species of the Fasciolidae (*Fasciola hepatica* Linneuus, 1758, *Fasciola gigantica* Cobbold, 1856, and *Fasciola* sp. GHL-2014) (Liu et al. 2014a; Chen et al. 2016; Liu et al. 2016); the gene atp8 is similarly missing, as usual in trematode species. All genes are transcribed in the anticlockwise direction and encoded by H strand (Table 1), which is in accordance with other digeneans. The mt genome of *A. hwangtsiyui* has 22 intergenic spacers ranging from 1 to 15 bp and contains two overlapping nucleotides ranging from 1 to 40 bp (Table 1). Noteworthily, a 40 bp overlap between the nad4 and nad4L genes exists in the *A. hwangtsiyui* mitogenome, which is consistent with most helminths such as *Eurytrema pancreaticum* Janson, 1889 (Chang et al. 2016), *Hypoderaeum conoideum* (Bloch, 1782) (Yang et al. 2015), but shorter than that of *Schistosoma mekongi* Voge, Bruckner & Bruce, 1978 (64 bp; Littlewood et al. 2006). The nucleotide contents of T, C, A, G, in *A. hwangtsiyui* mitogenome are 42.8%, 12.0%, 16.8%, and 28.5%, respectively (Table 2). The whole A+T content of the mitogenome is 59.6%, which was markedly biased toward T over A (AT skew: –0.437), and G over C (GC skew: 0.408).

**Table 1.** The organization of the mitochondrial genome of *Azygia hwangtsiyui*.

Gene	Position		Size	Intergenic nucleotides	Codon		Anti-codon	Strand
	From	To			Start	Stop		
cox3	1	660	660	–	ATG	TAG	–	H
trnH	666	729	64	+5	–	–	GTG	H
cytb	732	1841	1110	+2	ATG	TAG	–	H
nad4L	1848	2108	261	+6	ATG	TAG	–	H
nad4	2069	3340	1272	–40	ATG	TAG	–	H
trnQ	3345	3409	65	+4	–	–	TTG	H
trnF	3423	3488	66	+13	–	–	GAA	H
trnM	3490	3555	66	+1	–	–	CAT	H
atp6	3556	4068	513	–	ATG	TAG	–	H
nad2	4072	4932	861	+3	GTG	TAG	–	H
trnV	4946	5009	64	+13	–	–	TAC	H
trnA	5013	5076	64	+3	–	–	TGC	H
trnD	5081	5146	66	+4	–	–	GTC	H
nad1	5149	6054	906	+2	GTG	TAG	–	H
trnN	6070	6134	65	+15	–	–	GTT	H
trnP	6148	6212	65	+13	–	–	TGG	H
trnI	6216	6279	64	+3	–	–	GAT	H
trnK	6280	6348	69	–	–	–	CTT	H
nad3	6349	6708	360	–	ATG	TAA	–	H
trnS1	6712	6770	59	+3	–	–	GCT	H
trnW	6781	6842	62	+10	–	–	TCA	H
cox1	6843	8396	1554	–	TTG	TAG	–	H
trnT	8410	8474	65	+13	–	–	TGT	H
rrnL	8475	9449	975	–	–	–	–	H
trnC	9450	9506	57	–	–	–	GCA	H
rrnS	9507	10246	740	–		–	–	H
cox2	10247	10828	582	–	GTG	TAA	–	H
nad6	10834	11277	444	+5	GTG	TAG	–	H
trnY	11284	11352	69	+6	–	–	GTA	H
trnL1	11352	11416	65	–1	–	–	TAG	H
trnS2	11421	11490	70	+4	–	–	TGA	H
trnL2	11491	11555	65	–	–	–	TAA	H
trnR	11558	11617	60	+2	–	–	TCG	H
nad5	11626	13225	1600	+8	GTG	T	–	H
trnE	13226	13288	63	–	–	–	TTC	H
trnG	13604	13669	66	–	–	–	TCC	H

**Table 2.** Nucleotide contents of genes and the non–coding region within the mitochondrial genome of *Azygia hwangtsiyui*.

Regions	Size (bp)	T	C	A	G	AT (%)	GC (%)	AT skew	GC skew
atp6	513	50.5	11.3	12.9	25.3	63.4	36.6	–0.594	0.383
cox1	1554	44.0	13.0	15.9	27.1	59.9	40.1	–0.469	0.352
cox2	582	39.7	13.1	18.0	29.2	57.7	42.3	–0.375	0.382
cox3	660	44.8	11.4	15.9	27.9	60.7	39.3	–0.476	0.421
cytb	1110	44.4	13.5	15.8	26.3	60.2	39.8	–0.476	0.321
nad1	906	44.0	10.0	16.3	29.6	60.3	39.6	–0.459	0.493
nad2	861	46.2	11.3	11.8	30.7	58.0	42.0	–0.592	0.463
nad3	360	45.8	8.1	14.2	31.9	60.0	40.0	–0.528	0.597
nad4	1272	45.7	12.8	12.3	29.2	58.0	42.0	–0.577	0.391
nad4L	264	47.0	8.0	17.8	27.3	64.8	35.3	–0.450	0.548
nad5	1600	46.4	9.5	14.1	30.0	60.5	39.5	–0.535	0.519
nad6	444	45.9	11.9	14.2	27.9	60.1	39.8	–0.528	0.401
rrnL	975	37.4	14.1	22.5	26.1	59.9	40.2	–0.250	0.299
rrnS	740	36.6	14.5	21.6	27.3	58.2	41.8	–0.258	0.307
tRNAs	1349	35.3	14.5	21.7	28.5	57.0	43.0	–0.238	0.328
rRNAs	1715	37.1	14.2	22.1	26.6	59.2	40.8	–0.253	0.303
PCGs	10126	45.2	11.5	14.7	28.6	59.9	40.1	–0.509	0.425
Genome	13973	42.8	12.0	16.8	28.5	59.6	40.5	–0.437	0.408

### Protein-coding genes and non-coding regions

A total of 3364 amino acids was encoded by the *A. hwangtsiyui* mtDNA. The full scale of 12 concatenated protein-coding genes was 10126 bp, composed of 45.2% T, 11.5% C, 14.7% A, and 28.6% G. Average A+T content of concatenated 12 protein-coding genes was 59.9%, varying from 57.7% (cox2) to 64.8% (nad4L) (Table 2 and Suppl. material 2: Table S2). All 12 protein-coding genes of *A. hwangtsiyui* mt genome have a lower A+T percentage than those of *Trichobilharzia szidati* Neuhaus, 1952, *Calicophoron microbothrioides* Price & McIntosh, 1944, and some members of the Schistosomatidae Poche, 1907, but possess a higher A+T percentage than those of *Metagonimus yokogawai* Katsurada, 1912, and *Paragonimus westermani* Kerbert, 1878 (Suppl. material 3: Table S3) (Lee et al. 1987; Littlewood et al. 2006; Biswal et al. 2014; Semyenova et al. 2017; Oey et al. 2019). There is an obvious bias towards T over A (AT skew = –0.509), and G over C (GC skew = 0.425), and the coding strand is enriched with T and poor with A and especially C. For *A. hwangtsiyui*, the length of protein-coding genes was followed in the order: nad5 (1600 bp) > cox1 (1554 bp) > nad4 (1272 bp) > cytb (1110 bp) > nad1 (906 bp) > nad2 (861 bp) > cox3 (660 bp) > cox2 (582 bp) > atp6 (513 bp) > nad6 (444 bp) > nad3 (360 bp) > nad4L (264 bp). There are two non-coding regions (NCR1 and NCR2) in *A. hwangtsiyui* mitogenome, while the mt genome of *Paragonimus heterotremus* Chen et Hsia (1964), *C. complanatum*, *Fascioloides magna* (Bassi, 1875), and *T. szidati* have a single non-coding region (Chen et al. 2016; Ma et al. 2016; Semyenova et al. 2017; Qian et al. 2018). NCR1 and NCR2 of the *A. hwangtsiyui* mitogenome is partitioned into two parts by trnG, and accompanied by 70.5% and 57.6% A+T content, respectively. NCR1 and NCR2 have similar chemical base counts, 315 bp and 317 bp in size, respectively. While the NCR1 lacks distinguishing features and any tandem repeats, the NCR2 contains two typical tandem repeats, and each of tandem repeats sequence (120 bp) forms a hairpin-like secondary structure including a whole set of stems and loops (Suppl. material 4: Figure S1). Although tandem repeats are a segment of function-deficiency mitochondrial genome sequences, its hairpin-like secondary structures are widely perceived as regulating the replication and transcription of mitochondrial genome.

### Codon usage, transfer RNAs, and ribosomal RNAs

For the *A. hwangtsiyui* mitogenome, codon ends in G or T were more continual than those ending in A or C. The most frequently used start codon in protein-coding genes was ATG (for six PCGs), secondly was GTG (for five PCGs), which resembles that of the most frequent extrapolated start codons for mitogenome protein-encoding genes of digenean species (Chen et al. 2016). The least-used start codon was TTG (only one PCGs). Likewise, the most-used terminal codon was TAG (for nine PCGs), followed by TAA (for two PCGs). Only one of 12 protein-coding sequence (nad5) was terminated with abbreviated T stop codon (Table 1). Although incomplete stop codons (T or TA) frequently occur in cestodes and nematodes, they were rarely presented in flukes other than *D. chinensis*, *Dicrocoelium dendriticum* (Rudolphi, 1819), and *Postharmostomum commutatum* (Dietz, 1858) (Liu et al. 2014b; Fu et al. 2019). The codon UUU (Phe, 10.17%), UUG (Leu, 8.17%), and GUU (Val, 7.19%) were the most frequently occurring codons in protein-coding genes. Leucine, valine, and phenylalanine are the most-used amino acids, with frequency of 15.96%, 13.38%, and 11.09%, respectively. The least-used codons were CGA (Arg, 0.06%) and GAC (Asp, 0.15%), and the least frequent utilized amino acid was glutamine (1.01%) (Suppl. material 5: Figure S2). As most of digenea mitogenome sequences, the mitogenome of *A. hwangtsiyui* possessed 22 commonly found tRNAs, with the exception of that of *P. westermani* Korean isolate (23 tRNAs), and *P. westermani* Indian isolate (24 tRNAs) (Biswal et al. 2014). In *A. hwangtsiyui*, tRNA-Gly (trnG) is located between NCR1 and NCR2 (Fig. 1). The size of ribosomal RNA genes (rrnL and rrnS) in mitochondrial DNA of *A. hwangtsiyui* are 975 bp and 740 bp, respectively (Table 2). The upstream and downstream of rrnL and rrnS are cascaded with trnT and cox2 genes, respectively, and are detached from each other by trnC, as in all reported platyhelminths to date (Littlewood et al. 2006, Le et al. 2016).

### Gene arrangement

**Figure 2.**
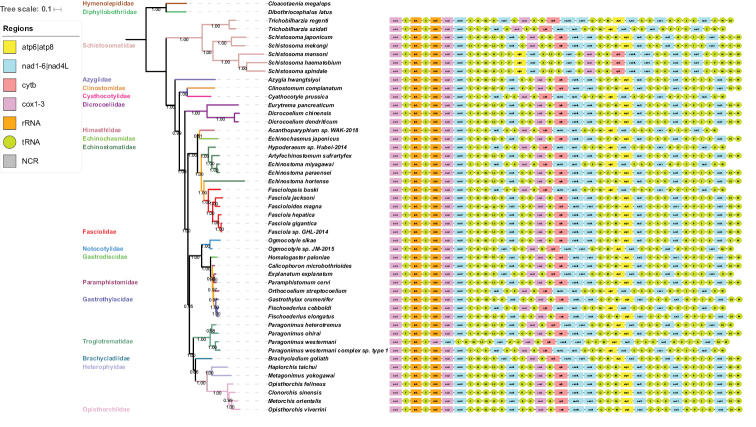
Phylogenetic relationships and gene arrangement of *Azygia hwangtsiyui* with other selected digeneas based on translated mitochondrial proteins. The concatenated amino-acid sequence datasets of the 12 protein-coding genes were analyzed by Bayesian Inference (BI) and Maximum Likelihood (ML), utilizing *Cloacotaenia megalops* (NC_032295.1) and *Dibothriocephalus latus* (NC_008945.1) as the outgroups. Both ML and BI analyses constructed identical tree topologies.

Comparative analysis of gene arrangement among 47 selected digenean taxa, two gene blocks (cox1-trnT-rrnL-trnC-rrnS-cox2-nad6 and cytb-nad4L-nad4-trnQ) are shared by all selected taxa (Fig. 2). Disregarding *P. heterotremus* and members of the family Schistosomatidae and Fasciolidae Raillet, 1895, the gene order of the remaining digenea taxa is virtually identical with the exception of the translocation of trnE and trnG among the remaining members of selected digenea representatives in family level. Intriguingly, there is the translocation of trnE and trnG within different species of family Fasciolidae. The translocations of three tRNAs (trnS1, trnS2 and trnS) can be discovered even between taxa of the same subgroup. Gene order of the *Brachycladium goliath* (Van Beneden, 1858) mt genome (the only representative of family Brachycladiidae Faust, 1929) is nearly same as that of *P. westermani* (Troglotrematidae Ward, 1918) except for the relocations of trnY between trnG and cox3, and trnE to the position between trnN and trnP. The groups of Schistosomatidae show a massive gene reorganization of protein-coding genes and tRNAs compared with other sequenced digenea mitogenome, which is in accord with previous finding reported by Littlewood et al. (2006).

### Mitogenome-derived phylogeny

To assess phylogenetic relationships among available flatworms, we utilized concatenated amino acid sequence dataset representing 12 protein-coding genes of *A. hwangtsiyui*, 46 other digenean representatives, and two tapeworm species (*C. megalops* and *D. latus*) for analyzing molecular-based phylogeny. In this study, the topological structure is divided into two large clades: one consists of seven members of the family Schistosomatidae; and the other clade comprises 40 members from 16 families including the family Azygiidae (*A. hwangtsiyui*) (Fig. 2). The topological structure shows that *A. hwangtsiyui* (Azygiidae) is identified as the most basal lineage of the Digenea, but separated from *C. complanatum* (Clinostomidae Lühe, 1901), and *Cyathocotyle prussica* Mühling, 1896 (Cyathocotylidae Poche, 1926). Phylogenetic analyses of all complete digenea mtDNAs confirmed taxonomic and previous phylogenetic assessments (Olson et al. 2003; Kostadinova and PéRez-del-Olmo 2014; Fu et al. 2019). The intricate structure and varying content of the family Azygiidae still awaits investigation of relationships based on a much wider taxon sampling and more mitogenome datasets.

## Supplementary materials

Supplementary material 1 Table S1 Data type molecular data Explanation note Primers for amplification and sequencing mitochondrial genome of *Azygia hwangtsiyui*. https://binary.pensoft.net/file/428057This dataset is made available under the Open Database License (http://opendatacommons.org/licenses/odbl/1.0/). The Open Database License (ODbL) is a license agreement intended to allow users to freely share, modify, and use this Dataset while maintaining this same freedom for others, provided that the original source and author(s) are credited.Yuan-An Wu, Jin-Wei Gao, Xiao-Fei Cheng, Min Xie, Xi-Ping Yuan, Dong Liu, Rui Song

Supplementary material 2 Table S2 Data type molecular data Explanation note Information of the Digenea and the outgroups for which complete mitogenomes are available in GenBank. https://binary.pensoft.net/file/428058This dataset is made available under the Open Database License (http://opendatacommons.org/licenses/odbl/1.0/). The Open Database License (ODbL) is a license agreement intended to allow users to freely share, modify, and use this Dataset while maintaining this same freedom for others, provided that the original source and author(s) are credited.Yuan-An Wu, Jin-Wei Gao, Xiao-Fei Cheng, Min Xie, Xi-Ping Yuan, Dong Liu, Rui Song

Supplementary material 3 Table S3 Data type molecular data Explanation note A+T content (%) for 12 protein-coding genes in the available 49 Platyhelminthes mitogenomes. https://binary.pensoft.net/file/428059This dataset is made available under the Open Database License (http://opendatacommons.org/licenses/odbl/1.0/). The Open Database License (ODbL) is a license agreement intended to allow users to freely share, modify, and use this Dataset while maintaining this same freedom for others, provided that the original source and author(s) are credited.Yuan-An Wu, Jin-Wei Gao, Xiao-Fei Cheng, Min Xie, Xi-Ping Yuan, Dong Liu, Rui Song

Supplementary material 4 Figure S1 Data type molecular data Explanation note Secondary structure of tandem repeats in non-coding region 2 (NCR2) of *Azygia hwangtsiyui* mitochondrial genome. https://binary.pensoft.net/file/428060This dataset is made available under the Open Database License (http://opendatacommons.org/licenses/odbl/1.0/). The Open Database License (ODbL) is a license agreement intended to allow users to freely share, modify, and use this Dataset while maintaining this same freedom for others, provided that the original source and author(s) are credited.Yuan-An Wu, Jin-Wei Gao, Xiao-Fei Cheng, Min Xie, Xi-Ping Yuan, Dong Liu, Rui Song

Supplementary material 5 Figure S2 Data type molecular data Explanation note Relative synonymous codon usage (RSCU) of *Azygia hwangtsiyui* mitochondrial genome. https://binary.pensoft.net/file/428061This dataset is made available under the Open Database License (http://opendatacommons.org/licenses/odbl/1.0/). The Open Database License (ODbL) is a license agreement intended to allow users to freely share, modify, and use this Dataset while maintaining this same freedom for others, provided that the original source and author(s) are credited.Yuan-An Wu, Jin-Wei Gao, Xiao-Fei Cheng, Min Xie, Xi-Ping Yuan, Dong Liu, Rui Song
