# Supplementary figures and images for: Characterization and comparative analysis of the complete mitochondrial genome of Azygia hwangtsiyui Tsin, 1933 (Digenea), the first for a member of the family Azygiidae
Source: Zookeys. 2020 Jul 3;945:1–16. doi: 10.3897/zookeys.945.49681 (PMC7351859; doi:10.3897/zookeys.945.49681)

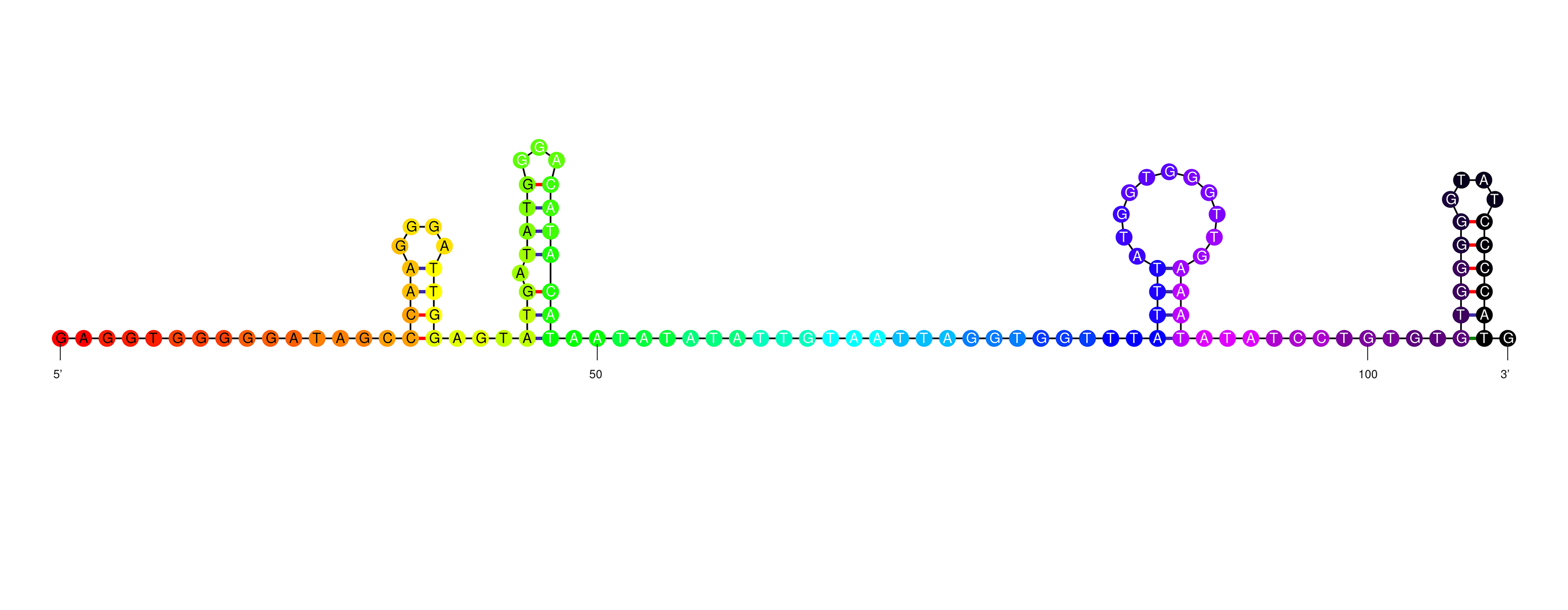

Supplement: Supplementary material 4 — Figure S1 [file zookeys-945-001-s004.tif]

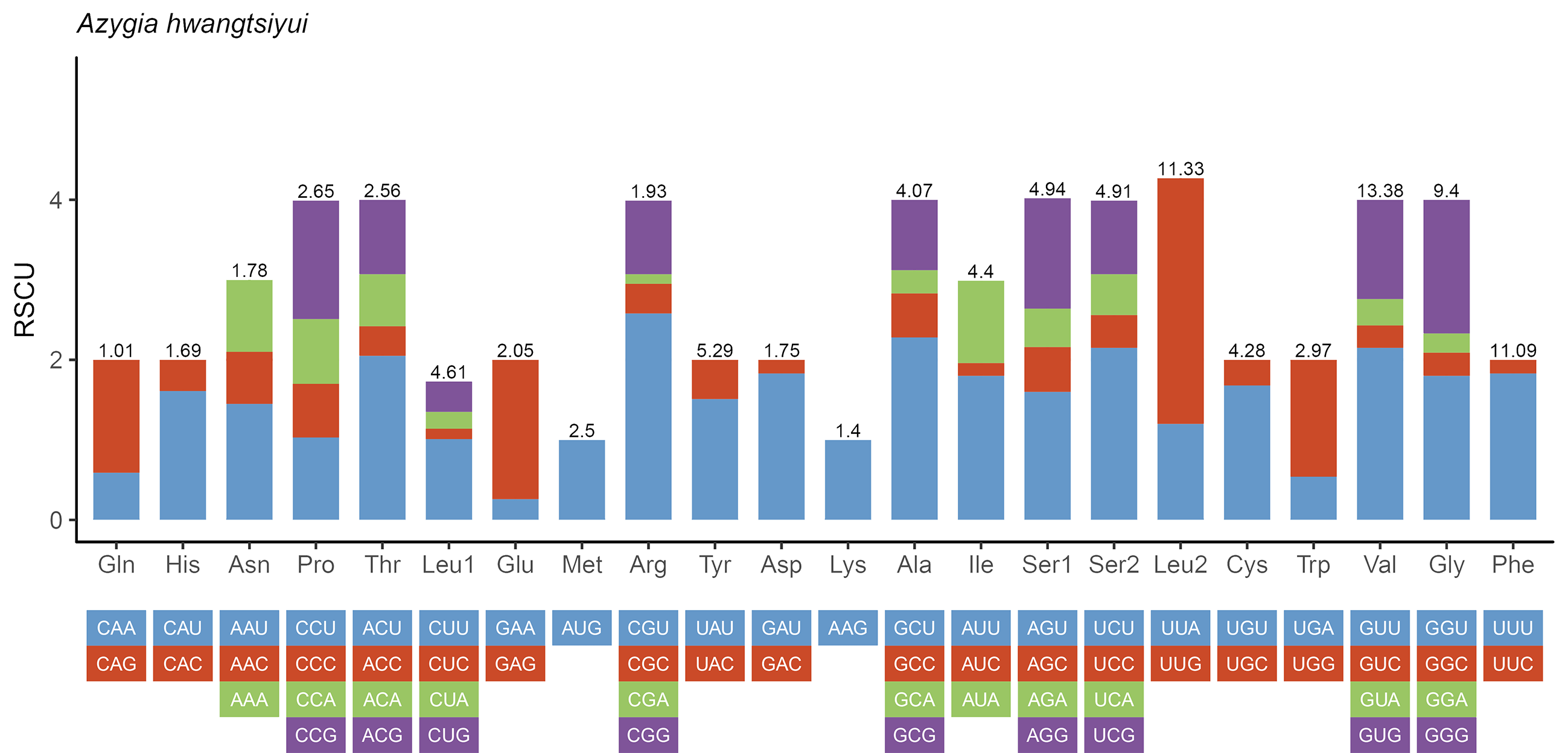

Supplement: Supplementary material 5 — Figure S2 [file zookeys-945-001-s005.tif]
